# Atomic layer deposition of vanadium oxide films for crystalline silicon solar cells†

**DOI:** 10.1039/d1ma00812a

**Published:** 2021-10-12

**Authors:** Eloi Ros Costals, Gerard Masmitjà, Estefania Almache, Benjamin Pusay, Kunal Tiwari, Edgardo Saucedo, C. Justin Raj, Byung Chul Kim, Joaquim Puigdollers, Isidro Martin, Cristobal Voz, Pablo Ortega

**Affiliations:** Electronic Engineering Department, Universitat Politècnica de Catalunya (UPC) 08034 Barcelona Spain eloi.ros@upc.edu; Department of Advanced Materials for Energy, Catalonia Institute for Energy Research (IREC) 08930 Barcelona Spain; Department of Chemistry, Dongguk University-Seoul Jung-gu Seoul-04620 Republic of Korea; Department of Advanced Components and Materials Engineering, Sunchon National University 255, Jungang-ro, Sunchen-si Jellanamdo 57922 Republic of Korea

## Abstract

Transition metal oxides (TMOs) are promising materials to develop selective contacts on high-efficiency crystalline silicon solar cells. Nevertheless, the standard deposition technique used for TMOs is thermal evaporation, which could add potential scalability problems to industrial photovoltaic fabrication processes. As an alternative, atomic layer deposition (ALD) is a thin film deposition technique already used for dielectric deposition in the semiconductor device industry that has a straightforward up scalable design. This work reports the results of vanadium oxide (V_2_O_5_) films deposited by ALD acting as a hole-selective contact for n-type crystalline silicon (c-Si) solar cell frontal transparent contact without the additional PECVD passivating layer. A reasonable specific contact resistance of 100 mΩ cm^2^ was measured by the transfer length method. In addition, measurements suggest the presence of an inversion layer at the c-Si/V_2_O_5_ interface with a sheet resistance of 15 kΩ sq^−1^. The strong band bending induced at the c-Si surface was confirmed through capacitance–voltage measurements with a built-in voltage value of 683 mV. Besides low contact resistance, vanadium oxide films provide excellent surface passivation with effective lifetime values of up to 800 μs. Finally, proof-of-concept both-side contacted solar cells exhibit efficiencies beyond 18%, shedding light on the possibilities of TMOs deposited by the atomic layer deposition technique.

## Abstract 2

We have fabricated n-type silicon solar cells using ALD deposited vanadium oxide as the front transparent contact.

## Graphical abstract

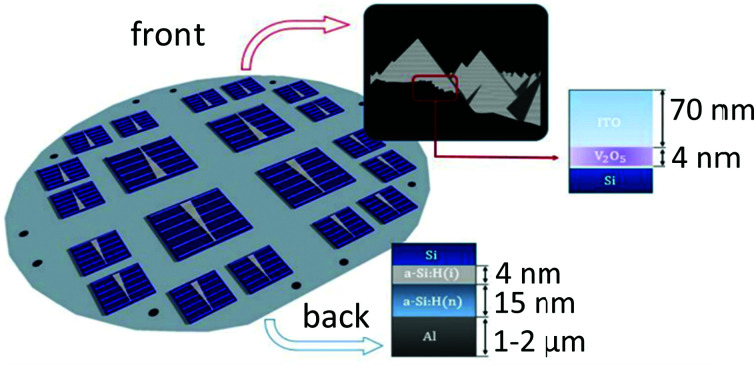

## Introduction

Since the launch of silicon photovoltaics (PV), an outstanding and continued improvement of all aspects of the value chain, such as materials, devices and manufacturing process have dramatically reduced fabrication costs and pumped silicon technology (crystalline and multicrystalline silicon) to up to 90.9% in the real market share of photovoltaic devices.^1^ As a consequence, solar PV have rapidly become one of the cheapest electricity sources all over the electrical market with a current levelized cost of electricity (LCOE) of around 2¢ kW h^−1^,^2^ indicating that solar energy harvesting is already a competitive alternative source of electricity to conventional combustion electric power plants.^3,4^

Current commercial silicon PV are generally based on homojunctions that involve high thermal stages, *i.e.*, diffusion processes to dope n^+^ and p^+^ regions beneath contacts. This approach provides tuning of the Fermi level in the material bulk that enhances selective charge collection and avoids unpleasant effects, such as Fermi-level pinning at the surfaces. For instance, structures based on the well-known Back Surface Field (BSF) and PERC technologies achieve relatively high efficiencies (*e.g.*, 19–20% and 20–22%, respectively),^5^ and their mass production manufacturing process is the main reason for this dominant position.

Apart from this more industrial scenario, it must be mentioned that the most efficient silicon solar cells under 1 Sun-illumination consist of doped amorphous silicon heterojunction (SHJ) technology that provides both surface passivation, and carrier collection with very low recombination losses in the so-called *passivating contacts*. In particular, a crystalline silicon (c-Si) solar cell based on SHJ contacts showing an outstanding world record of 26.7% efficiency has been reported using an Interdigitated Back Contact (IBC) structure.^6,7^ Such high efficiencies are reached in part thanks to the material quality improvement achieved in silicon substrates in the past years, *i.e.*, high bulk lifetimes, conferring a crucial role to surface passivation (front and rear) to boost even more solar cell performance. In this regard, these passivating structures are finding their way into a mass production due to their clear superior performance as in the case of HIT^8^ and TOPCON.^9,10^

In recent years, novel materials inherited from organic, Perovskites and other emerging PV technologies, have been proved as alternative carrier collectors on silicon solar cells.^11–19^ These materials generally do not require intentional doping, and most of them provide good performance using thin-film deposition directly on top of the silicon surface at low temperatures, or onto a previously deposited passivating layer. This alternative way to generate/induce junctions or provide a good ohmic contact on silicon is generally known as selective contacts. In fact, a functional solar cell requires, apart from an absorber, where photogeneration of electron–hole pair occurs, a contact, which may only extract holes (*i.e.*, hole transport layer – HTL) and blocks the collection of electrons, whereas, an additional contact is required to extract electrons (*i.e.*, electron transport layer – ETL) blocking the collection of holes.^20–22^

In this context, HTLs typically use Transition Metal Oxides (TMOs), such as molybdenum, vanadium and tungsten oxides (*i.e.*, MoO_3_, V_2_O_5_ and WO_3_).^23–26^ Alternatively, contacts based on either alkaline salts (LiF_*x*_ or MgF_2_)^11,12^ or TMOs, such as titanium oxide (TiO_2_)^15,17,27^ are good ETL candidates.

One of the most interesting properties of these materials is their wide bandgap (*i.e.*, >3 eV). This specific feature of TMOs can reduce the amount of current loss due to light absorption at the front transparent electrode with respect to a typical amorphous silicon heterostructure. Moreover, the thin film deposition of TMOs does not require the use of either high-temperature processes or hazardous gas precursors, both drawbacks of the aforementioned technologies (*i.e.*, BSF, PERC, PERT, PERL and SHJ). Furthermore, high-efficiency solar cells are possible with this type of selective contact, since an impressive 23.5% efficiency has been reported.^28^

Nevertheless, the standard deposition technique used for transition metal oxides on silicon requires a thermal evaporation step. Even though thermal evaporation is used in some roll-to-roll metallization processes, it's poor scalability in large scale production is one of the limitations that is preventing TMOs to have a relevant impact at the industrial level. Thus, new and more industrially scalable deposition techniques must be implemented for TMO-based c-Si solar cells to step into the PV market. In this sense, the Atomic Layer Deposition (ALD) process provides soft and low-temperature deposition techniques compatible with solar cell fabrication, as well as, it allows conformal deposition of films with a higher degree of scalability to industrial production than the thermal evaporation process.

Previous group research activity demonstrated that thin evaporated vanadium pentoxide (V_2_O_5_) films can efficiently work as a hole-selective layer.^24,29,30^ Moreover, the superior surface passivation on silicon substrates provided by vanadium oxide films has the advantage to potentially overcome the need to use an amorphous silicon film as a passivating interlayer^31^ eliminating a fabrication step and reducing the overall cost. ALD-based vanadium oxide films, in all different phases, have already been studied in other fields (*e.g.*, catalysis^32^ and dielectric use^33^), and fairly recently in photovoltaics.^34^ In this novel work, *Yang et al.* investigated possibilities of this technique for vanadium oxide using it in combination with an amorphous silicon-passivating buffer, obtaining outstanding devices with up to 21.6% efficiencies. Other attempts to fabricate operative photovoltaic devices with ALD deposited TMOs have not been as successful^35,36^ indicating how ALD deposition of transition metal oxides is not an easy task.

The explored solar cell architecture by *Yang et al.* does not take advantage of the reported TMO enhancement in optical properties with respect to the conventional heterojunction structure using amorphous silicon.^26^ Therefore, in this work, we report the properties of ALD vanadium oxide films as hole-selective contacts for silicon substrates in combination with transparent ITO electrodes. Finally, the optimized films are applied in finished solar cells with relevant efficiencies.

## Results and discussion

**Fig. 1:**
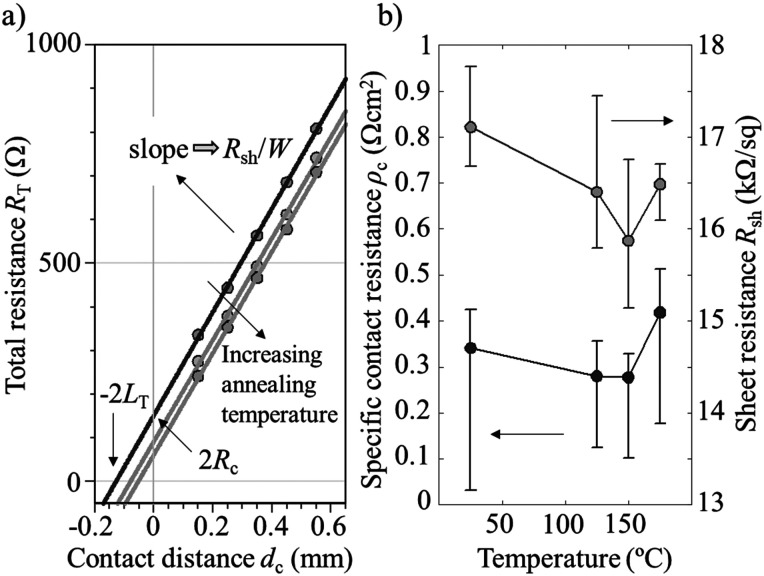
(a) Total resistance (*R*_T_) as a function of contact distance (*d*_c_) obtained from corresponding *I*–*V* curves for the ALD V_2_O_5_ film contacted with ITO/Ag. (b) Specific contact resistivity (*ρ*_c_) and Sheet resistance (*R*_sh_) dependence on annealing temperature extracted from TLM measurements.

The specific contact resistance (*ρ*_c_) and sheet resistance (*R*_sh_) of the V_2_O_5_/indium-tin-oxide (ITO) stacks on the c-Si(n) substrate were determined just after the deposition, using the transfer length method (TLM), resulting in average values of 340 mΩ cm^2^ and 17 kΩ sq^−1^, respectively (see Fig. 1b). It is important to note that very low *ρ*_c_ values below 40 mΩ cm^2^ have been measured in some samples. The relatively low extracted *R*_sh_ value corroborates the presence of an inverted p^+^ region at the silicon surface beneath the V_2_O_5_ layer as already reported in the literature using a V_2_O_5_/Au thermal-evaporated scheme.^37^ In general, within experimental error, the specific contact resistance and sheet resistance values are correlated; the lower the measured *R*_sh_ the lower is the *ρ*_c_. This result suggests that the specific contact resistance is improved when the hole concentration increases at the V_2_O_5_/c-Si interface, which could be expected.

Furthermore, the specific contact resistance exhibits a temperature dependence as can be seen in Fig. 1b, where cumulative 10 min annealing steps were made from 100 to 175 °C. The lowest average specific contact resistance value reached in the study corresponds to a 150 °C annealing treatment, reaching a value of 277 mΩ cm^2^ and a sheet resistance of 15.8 kΩ sq^−1^. In this case, a minimum and maximum *ρ*_c_ values of 100 and 430 mΩ cm^2^ were achieved, respectively, with *R*_sh_ ranging from 15 to 17 kΩ sq^−1^.

**Fig. 2:**
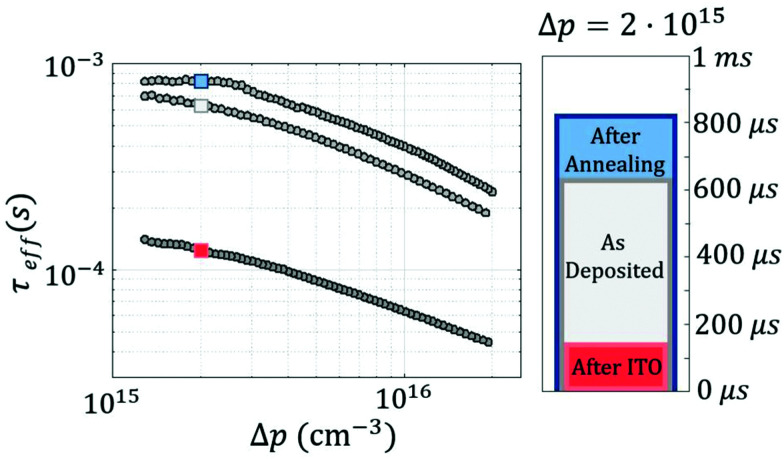
Effective carrier lifetime (*τ*_eff_) as a function of the excess carrier density (Δ*p*) of c-Si(n) substrate, with asymmetrically passivated front and back surfaces using ALD V_2_O_5_ and ALD Al_2_O_3_ films, respectively. Bar plot shows the *τ*_eff_ values at Δ*p* = 2 × 10^15^ cm^−3^.

From a passivation point of view, quasi-steady-state photoconductance (QSS-PC) measurements exhibit effective lifetimes (*τ*_eff_) of up to 600 μs for as-deposited samples, as can be seen in Fig. 2. After ITO deposition an important drop in surface passivation is observed decreasing lifetime to 140 μs. Damage after sputtering is a well-known phenomenon, damages, fortunately, can sometimes be recovered after a relatively low temperature annealing.^38^

It can be seen in Fig. 2, that annealing for 10 min at 150 °C totally recovered the sputtering damage and even enhanced a little more of the surface passivation, reaching *τ*_eff_ values of up to 820 μs. Such high lifetime results in implied open-circuit voltages as high as 690 mV for the finished device structure, and a recombination current density (*J*_0_) of 57.7 fA cm^−2^. This result indicates that evaporated and ALD vanadium films behave differently when exposed to thermal steps. Particularly, ALD deposited V_2_O_5_ films exhibited a positive response and a total recovery of sputtering damage.

**Fig. 3:**
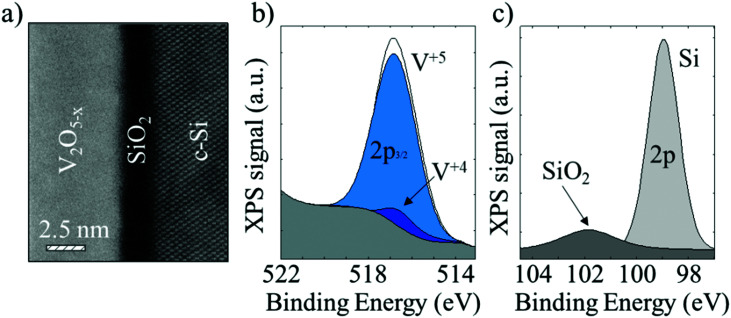
(a) A high-resolution STEM image of ALD V_2_O_5_ film deposited onto bare silicon, and its XPS spectra for the (b) vanadium 2p^3/2^ orbital and (c) silicon 2p orbital.

High-resolution scanning transmission electron microscope (STEM) images evidence the formation of a SiO_*x*_ interlayer between V_2_O_5_ and the crystalline silicon (see Fig. 3a). This spontaneous SiO_*x*_ growth layer at the silicon surface might explain in part the excellent surface passivation achieved in our films by reducing surface recombination states at the Si interface. Furthermore, X-ray photoelectron spectroscopy (XPS) data (see Fig. 3c) confirm the existence of a silicon oxide by analysing the 2p Si orbital spectrum.

This phenomenon also occurs using thermal evaporation as it has been already reported in the literature.^13^ In addition, in the 2p^3/2^ vanadium orbital a small presence of a sub-oxidized vanadium state (*i.e.*, V^4+^) is observed, which could be attributed to deeper gap states. These gap states are correlated to hole extraction through trap-assisted tunnelling.^39^ Furthermore, traps also contribute to the n-doping of the transition metal oxide layer. This feature is necessary to allow the band to band tunnelling of electrons from the conduction band of the vanadium oxide into the valence band of the crystalline silicon,^40,41^*i.e.*, the main hole extraction mechanism, which explains the relatively low contact resistance achieved in our samples.

**Fig. 4:**
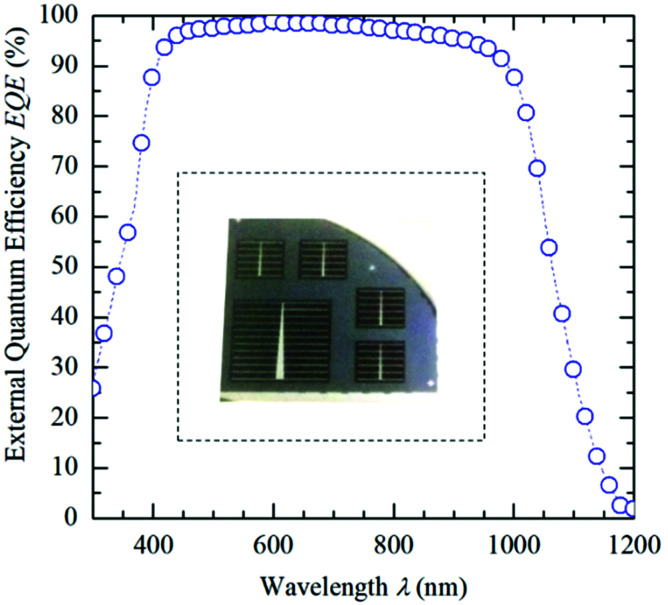
External quantum efficiency (EQE) curve of the best solar cell, in which the EQE beam spot was centered within the metallic fingers (*i.e.*, active device area). Inset: Photography showing solar cells of 1 × 1 and 2 × 2 cm^2^ configurations. IV curve and power output characteristics of the champion solar cell.

Finally, we fabricated proof-of-concept c-Si solar cells to demonstrate the viability of ALD V_2_O_5_ films as a hole-selective contact. The inset of Fig. 4 shows a photograph of finished devices. IV curves under AM 1.5G solar spectrum can be seen in Fig. 7 (1 kW m^−2^ at 25 °C) with a relatively high mean efficiency of 18.6 ± 0.24%, and an average open-circuit voltage (*V*_oc_), short circuit current density (*J*_sc_) and fill factor (FF) of 631 mV, 38.36 mA cm^−2^ and 75.8%, respectively. The external quantum efficiency (EQE) measurements (Fig. 4) confirmed that excellent front surface passivation is achieved in our cells with *J*_sc_ values higher than 38 mA cm^−2^ in all cases. On the other hand, *V*_oc_ values of up to 635 mV also confirm that vanadium oxide films provide enough front surface passivation and enhance the obtained *V*_oc_ in the order of 580/600 mV when compared to similar structures without an amorphous silicon buffer using evaporated TMOs.^18,41^ This could be due to the high degree of coverage of ALD.

The difference compared to the implied *V*_oc_ could be attributed to perimeter recombination (notice that in our solar cells there is no surface passivation outside active area), random pyramid surface, which increases the contact area and the non-negligible surface recombination at the rear contact.

From photovoltaic results summarized in Table 1, one can see that the vanadium oxide deposited by ALD can reach significantly high-efficiency solar cells even without the use of an additional interlayer in contrast to other reported works, where a thin interlayer of a-Si:H(i) is required to passivate the surface of c-Si.^30^

**Table 1:** Summary of the Solar cell parameters measured under dark conditions, standard light conditions (1 kW m^−2^, AM 1.5G spectrum, 25 °C) and Suns-*V*_oc_ measurements (Pseudo *J*–*V*, 1 Sun)

Area (cm^2^)	Dark *J*–*V*						AM 1.5G *J*–*V*				Pseudo *J*–*V*	
	*J*_01_ (pA cm^−2^)	*n*_1_	*J*_02_ (nA cm^−2^)	*n*_2_	*R*_s_ (Ω cm^2^)	*R*_shunt_ (MΩ cm^2^)	*V*_oc_ (mV)	*J*_sc_ (mA cm^−2^)	FF (%)	*η* (%)	pFF (%)	pη (%)
1	5.3	1.11	9.7	1.95	0.77	0.50	634	39.08	76.63	19.0	81.62	20.22
1	7.6	1.13	22	2.25	0.75	280	628	38.78	77.24	18.8	82.60	20.45
1	8.9	1.13	28.5	2.39	0.84	30	629	38.88	74.73	18.3	83.30	20.65
1	0.9	1.02	300	2.86	0.94	5	630	38.80	75.99	18.6	81.80	20.27
4	0.6	1.02	75	2.37	1.16	80	635	39.09	75.30	18.8	82.50	20.51
Mean ± std	5.0 ± 3.5	1.08 ± 0.06	150 ± 100	2.36 ± 0.32	0.89 ± 0.08	79 ± 50	631 ± 2.98	38.92 ± 0.11	75.82 ± 0.98	18.6 ± 0.24	82.36 ± 0.67	20.42 ± 0.17

In order to get a deeper insight into the V_2_O_5_/c-Si interface configuration, we measured capacitance–voltage curves of the solar cells in reverse bias. With this technique, we can determine the built-in voltage (*V*_bi_) of the junction, which corresponds to the induced band bending at the c-Si in thermal equilibrium.

This parameter is extracted from the *x*-axis crossing point of the *C*^−2^*vs.* voltage curve, known as the Mott–Schottky plot, plotted using the following eqn (1):^41^1
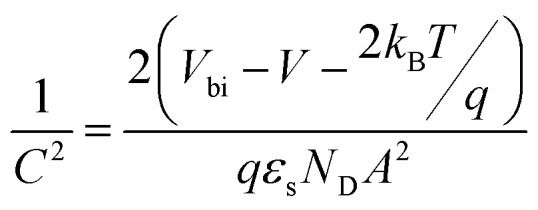
where *k*_B_ is the Boltzmann constant, *T* is the temperature, *q* is the fundamental charge, *ε*_s_ is the silicon dielectric constant and *N*_D_ is the doping density.

**Fig. 5:**
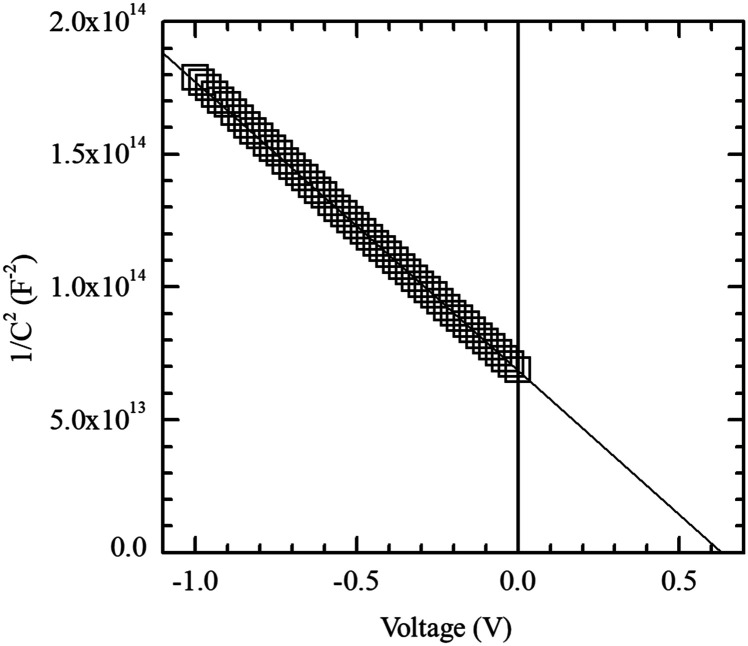
*C* ^−2^ *vs.* voltage plot (Mott–Schottky plot) of the 2 × 2 cm^2^ solar cell. From the *x*-axis crossing point, a *V*_bi_ of 683 mV is deduced indicating an inverted surface.

In Fig. 5, we show the *C*^−2^*vs.* voltage curve measured at 10 kHz for the 4 cm^2^ solar cell together with the best linear fit with *R*^2^ = 0.999925. From the slope of the linear fit, we can obtain the doping density of the substrate (*N*_D_ = 6.8 × 10^15^ cm^−3^, *ρ* = 0.75 Ω cm), which agrees well with the lower values of the resistivity range provided by the substrate manufacturer. In addition, a *V*_bi_ of 683 mV is extracted from the crossing point of the *x*-axis (*V* = 632 mV).

This band bending induced in c-Si corresponds to a surface with a very strong field-effect passivation. In other words, the surface shows a much higher hole density than electron density (inverted surface) significantly reducing interface recombination. Furthermore, this result confirms the presence of an inverted surface already suggested above by the TLM measurements.

**Fig. 6:**
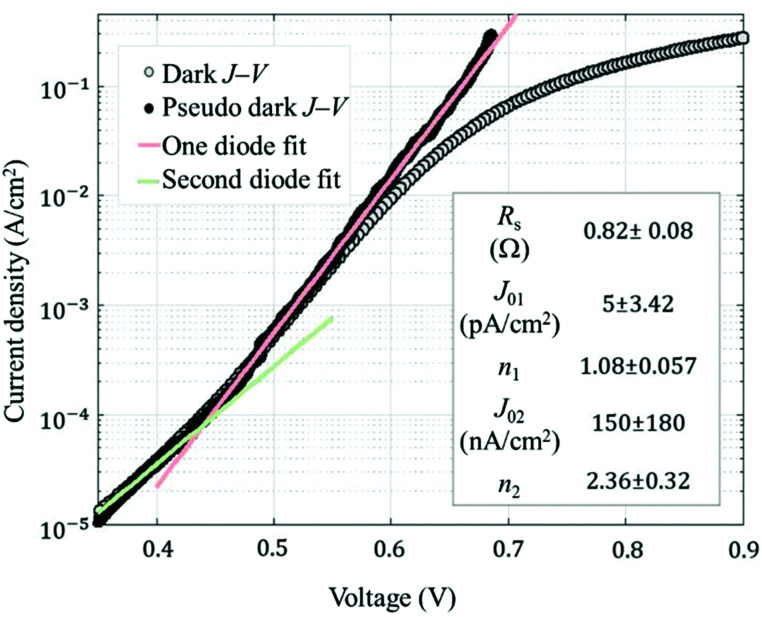
Dark *J*–*V* and pseudo dark *J*–*V* curves for the best solar cell. The pseudo *J*–*V* characteristic, free of series resistance effects, is determined from Suns-*V*_oc_ measurements. The fitting parameters are shown in the inset.

In order to determine the influence of the specific series resistance (*R*_s_) in the solar cell performance, Suns-*V*_oc_ measurements^42^ were made to extract the pseudo fill factor (pFF). The *R*_s_ parameter can be calculated using eqn (2) provided *V*_oc_, *J*_sc_ and pFF of the cell.^43^2
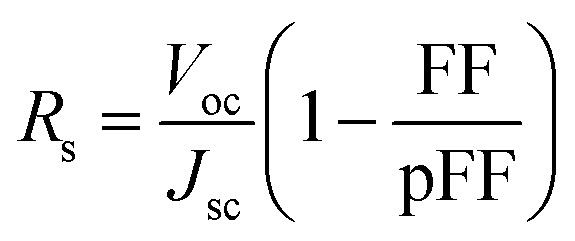
In this way, series resistance in our fabricated solar cells has an average value of 0.89 Ω cm^2^. Moreover, the pseudo-efficiency (p*η*) can also be calculated considering pFF, resulting in an average value of 20.42%. Additionally, we can extract dark parameters of measured devices, such as the recombination current density (*J*_0_) and diode ideality factor (*n*), by applying a two-diode model fitting, including both series and shunt resistance (*R*_shunt_)^44^ and using the expression given in eqn (3).3
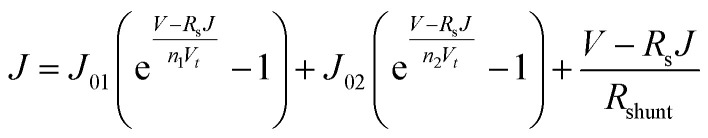
As an example, the fitting of the dark *J*–*V* characteristic of the best solar cell is shown in Fig. 6. A summary of all dark extracted parameters as well as the main photovoltaic parameters for each measured solar cell are summarized in Table 1. Interestingly, the ideality factor of the main diode is very close to unity (averaged 1.08), pointing out a good quality of the front heterojunction.

**Fig. 7:**
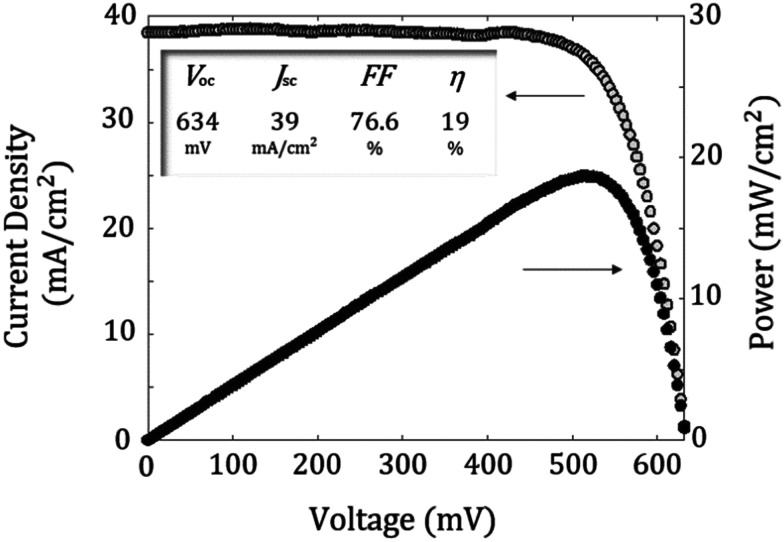
Light *J*–*V* and power output curves for the best solar cell. Photovoltaic parameters are shown in the inset.

By comparing pFF and FF values, we can conclude that the main mechanism that limits fill factor and efficiency is the series resistance, which is in the 0.75–1.16 Ω cm^2^ range. In order to elucidate the contribution of contact resistance of the V_2_O_5_-based HTL in our finished solar cells, we can apply eqn (4).^45^4
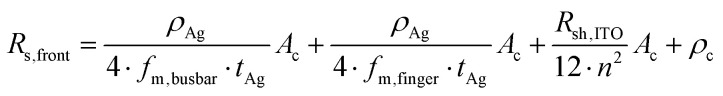
where *ρ*_Ag_, *t*_Ag_, *R*_sh,ITO_, *n*, *f*_m,busbar_ and *f*_m,finger_ are namely, the silver conductivity (1.59 μΩ cm), the thickness of the metallic grid (1.75 μm), the sheet resistance of the ITO layer (120 Ω sq^−1^), the number of fingers (6), and metallization factors due to busbar (1.6%) and fingers (1.6%), respectively. In this study, we have considered the solar cells with smaller area (*A*_c_ = 1 cm^2^), and we assume that in our fully back contacted devices, the whole series resistance (*R*_s_) is dominated by the front side term (*R*_s,front_) as a worst case, *i.e. R*_s_ ≅ *R*_s,front_.

Using eqn (4), we can extract *ρ*_c_ considering the average *R*_s_ value of 0.89 Ω cm^2^, reaching 0.33 Ω cm^2^ in our V_2_O_5_/ITO selective contacts deposited on the random pyramid textured surface. Curiously, this value is similar to that achieved in the TLM test samples on the polished surface despite of the increase in the contacted area due to the texturized surface. Notice that the contribution of *ρ*_c_ in the total resistance is below 37%, suggesting that there is still room to improve the fill factor, *i.e.*, using a metallization with higher thickness of Ag and/or reducing the sheet resistance of the ITO layer.

## Experimental

The test sample and solar cell devices were fabricated using high-quality 〈100〉 float zone n-type c-Si (c-Si) wafers of 4” with resistivity and thickness of 1.5 ± 1 Ω cm and 280 ± 20 μm, respectively. The specific contact resistance (*ρ*_c_) of ALD V_2_O_5_ selective contact was determined using transfer length measurements (TLM) summarized for contact resistivity as follows in eqn (5):^46,47^5
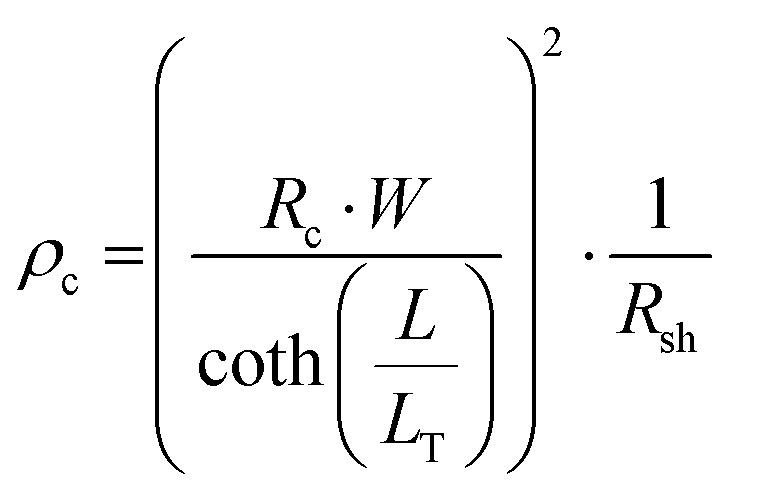
where *L* and *W* are the length and width of the metal contacts, respectively, *R*_sh_ is the sheet resistance of the semiconductor material through which the current between contacts flows. Transfer length (*L*_T_) and contact resistance (*R*_c_) parameters are extracted from the TLM measurements considering the half value of *x*- and *y*-axis crossing points of the total resistance *vs.* pad spacing plot, respectively.

**Fig. 8:**
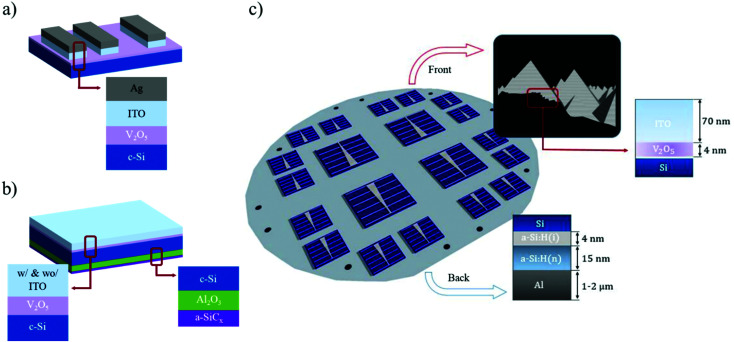
Schematic diagram of: (a) TLM film structure. (b) QSSPC film structure (c) solar cell film structure.

TLM samples (see Fig. 8a) were prepared on polished c-Si(n) wafers, which had been previously cleaned following standard RCA cleaning procedure,^48^ plus a diluted HF (1%) dip for 1 minute obtaining, as a result, hydrophobic silicon surfaces. Next, samples were introduced into the ALD system (Savannah S200, Cambridge Nanotech) and they were covered with a 4 nm thick V_2_O_5_ layer. Tetrakis(ethylmethylamino)-vanadium(iv) (*i.e.*, C_12_H_32_N_4_V) and deionized water (DI-H_2_O) were used as the vanadium precursor and oxidant species, respectively. The deposition process was performed at 125 °C and the vanadium precursor was heated at 58 °C, resulting in an estimated growth rate of 0.4 Å per cycle. Then, a 70 nm thick indium-tin-oxide (ITO) film was deposited using RF magnetron sputtering at 50 W, using a shadow-mask technique to pattern TLM contacts. Finally, without removing the shadow mask, a 200 nm thick silver layer was thermally evaporated over the ITO film. The dependence of contact resistance on thermal treatments was studied using 10 min hot plate annealing under nitrogen (N_2_) rich ambient.

Surface passivation properties of ALD V_2_O_5_ films were obtained by the effective lifetime (*τ*_eff_) measurements, using the quasi-steady-state photoconductance (QSS-PC) technique^49^ with a WCT-120 instrument (Sinton Consulting). Asymmetrical test samples were prepared for this purpose (see Fig. 8b), with a back-surface excellently passivated with an alumina film (*i.e.*, surface recombination velocity close to zero) and a front surface covered with the corresponding film under study, following the next steps: first, after RCA cleaning, a 50 nm thick ALD alumina film was deposited on both sides at a temperature of 200 °C. Trimethylaluminum (TMA) and DI-H_2_O were used as the aluminum precursor and oxidant species, respectively. A subsequent annealing treatment, 10 min in forming gas (FG) ambient at 400 °C, was made to activate surface passivation provided by alumina films.^50^ In order to protect the alumina film in the subsequent process etching stages, a 35 nm thick silicon carbide layer (a-SiC_*x*_) film was deposited over alumina on the backside using the plasma-enhanced chemical vapor deposition (PECVD) process (13.56 MHz, from Elettrorava S.p.A). This stage was made at 300 °C using methane (CH_4_) and silane (SiH_4_) as precursor gases in the PECVD reactor. Finally, after an HF (1%) dip to remove the front-side alumina film (*i.e.*, obtaining a bare hydrophobic c-Si surface) a 4 nm thick V_2_O_5_ film was deposited at 125 °C, using the same conditions described before for the TLM test samples. The passivation properties after ITO deposition were also studied, in which a 70 nm thick ITO layer was deposited over V_2_O_5_ using the same sputtering conditions for TLM test samples. Healing of any eventual damage by the sputtering process was performed with a final annealing treatment at 150 °C for 10 min on a hot plate in N_2_ ambient.

The surface composition of V_2_O_5_ films was determined using X-ray photoelectron spectroscopy (XPS) (SPECS, hemispherical energy analyzer PHOIBOS 150). The scan was performed using a non-monochromatic Al-Kα X-ray excitation source at 1486.6 eV and 3 × 10^−9^ mbar.

Current density *vs.* voltage (*J*–*V*) electrical characteristics of the complete devices were measured under a four-probe configuration using a Keithley 2601B Source Meter.

Solar cells of 1 × 1 and 2 × 2 cm^2^ active area were fabricated to demonstrate the viability of V_2_O_5_ films as a hole-selective contact. The device structure is depicted in Fig. 8c. The fabrication process started with the texturization of the front side c-Si(n) wafer with random pyramids by alkaline etching using tetramethylammonium hydroxide (TMAH) alkaline-based solution. Then, the substrate was cleaned by a standard RCA procedure and HF (1%) dip for 1 min, followed by the deposition of an intrinsic and a phosphorus-doped amorphous silicon stack [a-Si:H(i)/a-Si:H(n)] with thicknesses of 4 and 15 nm, respectively. The stack was deposited by the same foregoing PECVD equipment used for the test samples at a temperature of 300 °C. Next, just after another RCA cleaning process, the ALD V_2_O_5_ film (4 nm) was deposited on the front surface and immediately introduced into the sputtering chamber to deposit a 70 nm thick ITO layer at 50 W. Next, front active solar cell areas were defined using standard photolithography and wet etching (HF (2%) dip, 2 min). After that, a fully back-contact metallization was performed using a thermally evaporated Al film (1 μm) over the a-Si:H(i)/a-Si:H(n) stack. Finally, the front-contact silver grid (50 μm wide fingers) was thermally evaporated using a shadow-mask, resulting in a metal fraction area of around 4%. Final annealing in forming gas ambient at 150 °C was made to recover sputtering damage. Photovoltaic parameters and current–voltage curves were measured under standard test conditions AM 1.5G solar spectrum (1 kW m^−2^ at 25 °C) using an ORIEL 94021A (Newport) solar simulator, the light irradiance of which was properly calibrated using a pyranometer. External quantum efficiencies (EQE) curves were obtained using a commercial instrument (QEX10, PV measurements) with a white light bias of 0.2 Suns and a beam spot placed directly onto the ITO and out of the metallic fingers patterned on the front surface (*i.e.*, the active device area).

## Conclusions

In this work, we studied the vanadium oxide thin films deposited by ALD and studied their application as a hole transport layer in crystalline silicon solar cells as a transparent electrode without a PECVD buffer passivation layer.

TLM measurements of V_2_O_5_ (ALD)/ITO/Ag stacks on c-Si(n) confirm low contact resistances, reaching values as low as 40 mΩ cm^2^ with an average value of 0.4 Ω cm^2^. Furthermore, the observed sheet resistance of TMO is in the order of 15–20 kΩ sq^−1^ correlating to the presence of a p^+^ inversion layer beneath the film. Capacitance measurements on the stack also evidenced the presence of an inversion layer with a built-in voltage value of 683 mV. This inverted layer also provides both low contact resistance and good surface passivation with effective minority carrier lifetimes up to 800 μs after thermal treatment.

Finally, we tested ALD-deposited vanadium oxide as the HTL in c-Si solar cells. The fabricated cells measured under standard AM 1.5G solar spectrum (1 kW m^−2^, *T* = 25 °C) exhibited efficiencies of 18.6% on average, with mean values of *V*_oc_, *J*_sc_ and FF of 631 mV, 38.9 mA cm^−2^ and 75.8%, respectively. The low scattering between photovoltaic parameter values points out a remarkable reproducibility between devices, indicating a high layer conformal deposition over a four-inch wafer. The top solar cell achieved significant performance parameters with an efficiency of up to ∼19% without passivation from amorphous silicon. The main limiting factor in the final photovoltaic efficiency is the series resistance achieved by our finished device design corresponding to 0.89 Ω cm^2^ on average. However, the contribution of the V_2_O_5_-based front contact is estimated to be only 37% of the total series resistance. Therefore, technological improvements in cell fabrication, such as better electrical quality of the ITO layer (*R*_sh_ < 120 Ω sq^−1^) and/or a thicker silver grid could improve current efficiencies closer to the calculated pseudoefficiencies (>20%) and closer to those reported by Yang *et al.*,^34^ 21.6%, with an enhanced current provided by the use of wide bandgap materials in the front contact.

With the current range of efficiencies obtained in this study, we conclude that ALD is a suitable deposition technique for transition metal oxides as it is useful, particularly, for vanadium oxide on crystalline silicon for photovoltaic applications.

## Conflicts of interest

The authors declare that there are no conflicts of interest in regard of this manuscript.

## Supplementary Material

MA-003-D1MA00812A-s001
