# Supplementary figures and images for: Atomic layer deposition of vanadium oxide films for crystalline silicon solar cells
Source: Mater Adv. 2021 Oct 12;3(1):337–45. doi: 10.1039/d1ma00812a (PMC8724908; doi:10.1039/d1ma00812a)

### Supplementary information:

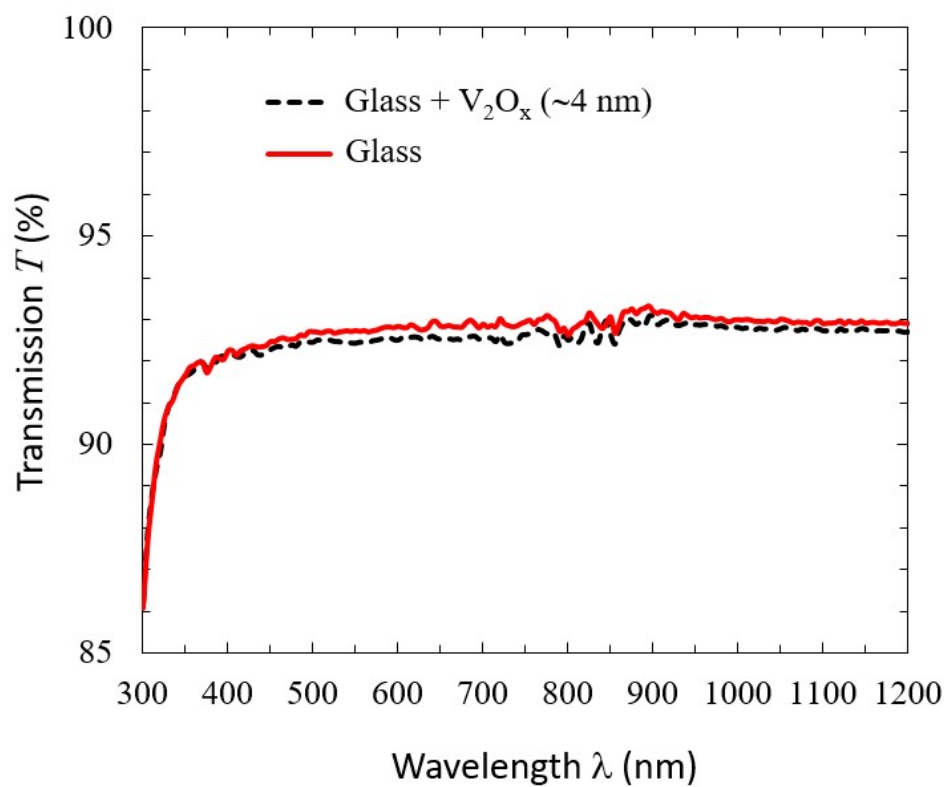

Supplement: MA-003-D1MA00812A-s001 [file MA-003-D1MA00812A-s001.pdf]
